# P-1451. Missed Opportunities for HIV Diagnosis in Mexico City: Unveiling the Gender Gap

**DOI:** 10.1093/ofid/ofae631.1623

**Published:** 2025-01-29

**Authors:** Nancy Sierra-Barajas, Yanink Caro-Vega, Juan G Sierra-Madero, Rodrigo Ville Benavides, Alvaro López-Iñiguez, Jessica Mejia-Castrejon, Karen Juarez-Campos, Angelina Silva-Casarrubias, Brenda Crabtree-Ramírez

**Affiliations:** Instituto Nacional de Ciencias Médicas y Nutrición Salvador Zubirán, Ciudad de México, Distrito Federal, Mexico; Instituto Nacional de Ciencias Medicas y Nutrición Salvador Zubirán, Tlalpan, Distrito Federal, Mexico; Instituto Nacional de Ciencias Médicas y Nutrición Salvador Zubirán, Ciudad de México, Distrito Federal, Mexico; Instituto Nacional de Ciencias Médicas y Nutrición "Salvador Zubirán", Tlalpan, Distrito Federal, Mexico; Instituto Nacional de Ciencias Médicas y Nutrición Salvador Zubirán., Mexico city, Distrito Federal, Mexico; Instituto Nacional de Ciencias Médicas y Nutrición Salvador Zubirán, Ciudad de México, Distrito Federal, Mexico; Instituto Nacional de Ciencias Médicas y Nutrición Salvador Zubirán, Ciudad de México, Distrito Federal, Mexico; Instituto Nacional de Ciencias Médicas y Nutrición Salvador Zubirán, Ciudad de México, Distrito Federal, Mexico; Infectious Diseases Department. Instituto Nacional de Ciencias Medicas y Nutrición Salvador Zubirán, Tlalpan, Distrito Federal, Mexico

## Abstract

**Background:**

Women in Mexico are not considered a key population for acquiring HIV, consequently, neither the patients nor their physicians are aware of this risk. This lack of recognition may contribute to late diagnoses and unfavorable clinical outcomes. This study aims to determine the frequency of missed opportunities for HIV diagnosis (MOHD) in a tertiary care center in Mexico City.

**Figure:**
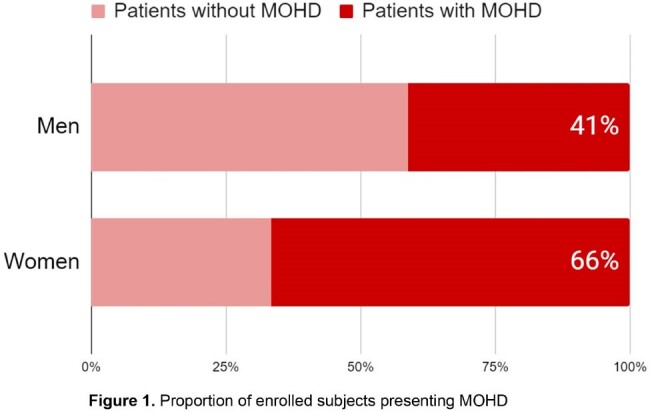

**Methods:**

A questionnaire for newly enrolled healthcare patients was routinely applied since 2013 to look for MOHD, at the HIV Clinic of a tertiary care center in Mexico City. MOHD was defined as a healthcare encounter in which a) HIV diagnosis was not made, b) was prompted by a clinical manifestation related to HIV, and c) occurred at least 30 days before the HIV diagnosis was confirmed. Frequency and proportion of MOHD by sex were reported, we described demographics, presence of AIDS at enrollment and compared the proportion of MOHD and AIDS defining events by gender.

**Figure:**
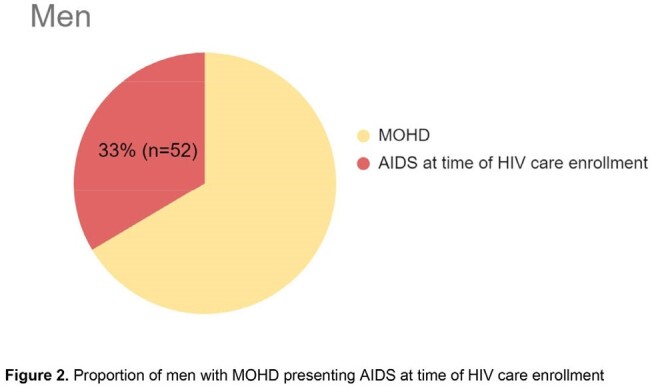

**Results:**

A total of 1,143 questionaries were applied from January 1^st^ 2013, to December 31^st^ 2023; 107 (9.4%) respondents were female and 1036 (90.6%) were male. A total of 486 (42.5%) patients reported experiencing symptoms related to HIV before their diagnosis, with 36 (7.4%) being women. 466 of 486 sought medical attention for their symptoms: 403 patients had data to establish MOHD, 27 were women and 376 men. MOHD was considered in 18/27 (66%) women and in 155/376 (41%) men (p=0.03). Among the patients with MOHD, 10/18 (56%) of the women and 52/155 (34%) of men had AIDS at enrollment in HIV care (p=0.02).

In the MOHD group, women's mean age was 43 years (SD=12.2) vs 35 in men (SD=12.2, p=0.01), 39% were married (7/18) vs. 11% of the men (17/155) (p< 0.01), and years of education were similar: 12.2 (SD= 4.6) vs. 13.2 (SD=4.2), respectively (p=0.51).

**Figure:**
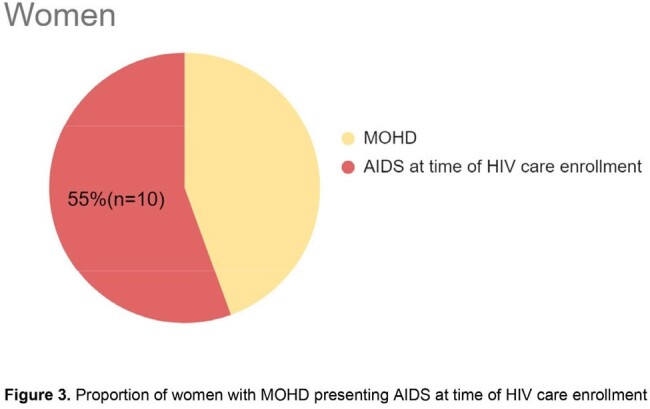

**Conclusion:**

This study reveals a higher proportion of missed opportunities for HIV diagnosis and AIDS in women compared to men. Women with MOHD were older and married in a higher proportion than men. Our results underscore the urgent need of strategies for increasing awareness of HIV risk in Mexican women.

**Figure:**
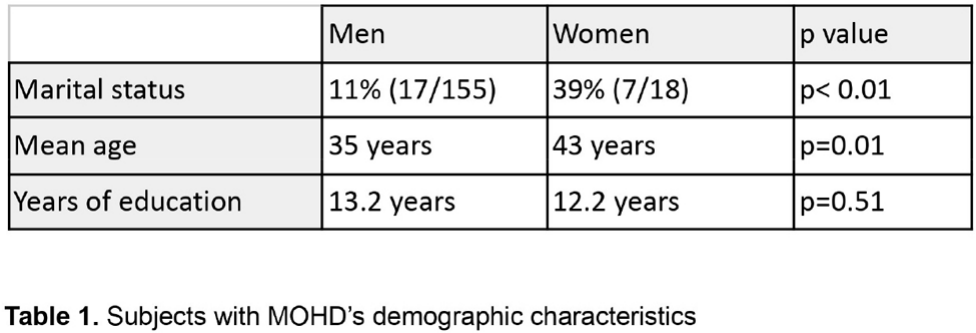

**Disclosures:**

**All Authors**: No reported disclosures

